# Caregiver Burden and Sleep Quality in Dependent People’s Family Caregivers

**DOI:** 10.3390/jcm8071072

**Published:** 2019-07-22

**Authors:** Miguel A. Simón, Ana M. Bueno, Patricia Otero, Vanessa Blanco, Fernando L. Vázquez

**Affiliations:** 1 Department of Psychology, University of A Coruña, 15701 A Coruña, Spain; 2 Department of Evolutionary and Educational Psychology, University of Santiago de Compostela, 15782 Santiago de Compostela, Spain; 3 Department of Clinical Psychology and Psychobiology, University of Santiago de Compostela, 15782 Santiago de Compostela, Spain

**Keywords:** caregiver burden, sleep quality, family caregivers, long-term care

## Abstract

This study examined the relationship between caregiver burden and sleep quality in dependent people’s family caregivers. A cross-sectional study was carried out with 201 dependent people’s family caregivers and 92 non-caregivers controls. Participants completed the Pittsburgh Sleep Quality Index (PSQI), the Caregiver Burden Inventory (CBI), and an ad-hoc questionnaire to collect sociodemographic data. Based on CBI scores, subjects were categorized into three groups: family caregivers with high levels of perceived burden, family caregivers with low and medium levels of perceived burden and non-caregiver controls. There were significant differences among the groups in the PSQI total (F = 40.39; *p* < 0.001), subjective sleep quality (F = 25.55; *p* < 0.001), sleep latency (F = 16.99; *p* < 0.001), sleep disturbances (F = 14.90; *p* < 0.001), use of sleep medications (F = 6.94; *p* < 0.01) and daytime dysfunction (F = 20.12; *p* < 0.001). These differences were found only between the caregivers with high levels of perceived burden and the other two groups (*p* < 0.05). There were also significant differences between the groups in sleep duration (F = 18.34; *p* < 0.001) and habitual sleep efficiency (F = 24.24; *p* < 0.001). In these dependent measures, the differences were found in all the pairs examined (*p* < 0.05). These results suggest that caregiver burden is related to sleep quality, so that caregivers with greater perceived burden have a worse sleep quality.

## 1. Introduction

Family caregivers are the most important informal caregivers that voluntarily provide support and unpaid care to a family member who cannot function with autonomy and independence. Caring for a dependent family member with a chronic disability is a very stressful task that can have adverse consequences for the health and health-related quality of life of the family caregiver [1,2]. These negative effects on the informal caregiver are closely related to what has been called “caregiver burden”. Caregiver burden is a complex and multidimensional construct, with subjective and objective components [3]; it has been defined as the physical, psychological, social and financial stressors experienced by caregivers due to the provision of care [4]. It is the product of the dynamic interaction among care needs of the dependent people, the care situation, and the caregiver’s resources and vulnerabilities.

Although it is very important to recognize that care can have powerful rewards and psychological benefits, due to the intensity of their care routines, the incessant realization of uncomfortable and exhausting tasks, and emotional reactions, such as feelings of guilt, dependent people’s family caregivers usually delay, neglect or do not seek medical help for their own health issues and they are more stressed than non-caregivers [5]. In fact, the prolonged care of dependent people can be one of the hardest experiences in a family caregiver’s life, resulting in very difficult to maintain adequate emotional balance. As a result, dependent people’s family caregivers have a high degree of psychological distress, particularly anxiety and depression [6,7,8,9]. Specially, caregivers who experience a high burden have an elevated psychological distress, presenting depression scores over twice as high as non-caregivers [10].

Family caregivers present impaired health behaviours and habits, as well as an increase in morbidity and mortality risk [11,12]. Studies consistently report a higher risk of cardiovascular disease [13], immunological disorders [14], and sleep difficulties and concerns, particularly sleep quality impairment and insomnia [15,16]. Specifically, it was found that family caregivers exhibit impaired quality of sleep compared to non-caregivers [17], and they had a prevalence of insomnia up to 41.0% [18]. The factors more strongly associated with this diagnosis were the years of care duration and the daily hours of care [18] as well as dementia-related behavioural disturbances in dementia caregivers [17].

The research carried out in the last years with dependent people’s family caregivers has evidenced that certain aspects of the caregiving experience, specially the informal caregiving intensity (measured by the type and/or quantity of care provided), is related to the amplitude of its effects on the quality of life and the physical and mental health of the caregiver [19,20,21]. That is to say, that the negative effects of caregiving on physical and mental health are most likely to be found in family caregivers providing high intensity care. Nevertheless, in the field of research on sleep in dependent people’s family caregivers, few studies have examined possible differences in sleep quality in the function of the caregiver burden [22,23,24]. Moreover, some of the studies carried out present certain methodological limitations, among which a small sample size and, mainly, the absence of a control group of non-caregivers stand out. This aspect is very important, because, with independence of the care situation, many different factors, such as age or gender, can explicate the poor sleep quality in family caregivers; these are significant issue, since most family caregivers are middle or advanced-age women [25].

The purpose of this cross-sectional study was to examine the relationship between caregiver burden and sleep quality in dependent people’s family caregivers. We predicted a direct relationship between caregiver burden and sleep quality, in such a way that family caregivers with high levels of perceived burden would evidence a worse sleep quality than family caregivers with low and medium levels of perceived burden and that of non-caregiver controls.

## 2. Materials and Methods

### 2.1. Subjects

The study sample comprised 201 dependent people’s family caregivers and 92 non-caregiver controls. Dependent people’s family caregivers were recruited by simple random sampling from the official register of caregivers prepared by the Dependency Technical Coordination Unit of the Ministry of Labour and Welfare of the Xunta de Galicia (Spain). All caregivers who were part of that registry cared for people whose degree of dependence ranged from moderate to total. To be eligible, caregivers were required to be a family caregiver (not a professional that charged for providing these services) of a person with officially recognized dependence and who lived in the same home (co-residence) as the cared person. Of the 210 family caregivers originally selected, 9 refused participation because they were too occupied (response rate of 95.7%), resulting in a final sample of 201 individuals, mostly women (87.1%), with a mean age of 56.2 years (SD = 10.1). The non-caregiver controls were persons not providing care to a household member. They were recruited by convenience sampling in the same proportions of gender, age and marital status as caregivers. Exclusion criteria of all participants (i.e., family caregivers and control subjects) were the presence of any major difficulty in communication that could interfere with the usual assessment procedures (e.g., severe visual impairment) and/or having received psychological or psychiatric treatment in the last two months.

The study was conducted according to the Declaration of Helsinki and was approved by the Bioethics Committee of the University of Santiago de Compostela (Code number 07092016). All participants gave written, informed consent. Participation was voluntary, without economic compensation or incentive of any kind.

### 2.2. Measures

An ad-hoc questionnaire was employed to collect data on the various sociodemographic characteristics of the family caregivers and the non-caregivers (i.e., age, gender, marital status, educational level, monthly incomes, family relationship with the dependent—the latter logically only in the case of family caregivers), of the care recipients (i.e., age, gender, cause of the dependence) and of the care situation (i.e., years providing care and daily hours of care).

The sleep quality of participants was assessed using the Spanish version of the Pittsburgh Sleep Quality Index (PSQI) [26] carried out by Royuela and Macías [27]. This instrument consists of 19 items, which are used to generate 7 subscales or components: subjective sleep quality, sleep latency, sleep duration, habitual sleep efficiency, sleep disturbances, use of sleep medications, and daytime dysfunction. Each subscale has a range of possible scores from 0 (very good) to 3 (very bad). The sum of these subscales scores yields the global PSQI score (range 0–21), with scores above 5 reflecting poor sleep quality and pathological difficulties in this area. The Spanish version of the PSQI has been found to have adequate psychometric properties, presenting an acceptable internal consistency, test–retest reliability, sensitivity, specificity, and predictive value, being a proper instrument for epidemiological and clinical research [27,28].

Family caregiver burden was measured with the Spanish version of the Caregiver Burden Inventory (CBI) [29] carried out by Vázquez et al. [30]. This 24 item multidimensional questionnaire quantifies the impact of burden on several domains of a caregiver’s life. Each question is rated using a 5 point Likert scale ranging from 0 (not at all descriptive) to 4 (very descriptive). The total score of the CBI ranges from 0 to 96, with higher scores indicating greater levels of perceived burden. This Spanish version presents a satisfactory internal consistency (Cronbach *α* = 0.89).

### 2.3. Procedure

Caregivers and controls were contacted through letters and phone calls. The characteristics of the study were explained to them and they were invited to participate. To minimize dropouts, data collection strategies for cross-sectional studies were followed [31], such as making the presentation of the study attractive to participants, treating the persons with kindness, affection and respect, reminding them of the date of the assessment and avoiding collecting information in an invasive way. Three previously trained psychologists carried out the assessment of the family caregivers and answered all questions that were raised. They collected the sociodemographic information of the caregivers, of the care recipients and of the care situation described above, and applied the questionnaires (i.e., PSQI and CBI) in a self-administered manner. Non-caregivers were recruited by convenience sampling from the Department of Psychology (University of A Coruña) and the Department of Clinical Psychology and Psychobiology (University of Santiago de Compostela) and they participated in an online assessment to obtain sociodemographic information and complete the PSQI.

All subjects were informed about the voluntary nature of their participation and the confidentiality of their responses, which should be honest and sincere. They were also informed about the possibility of leaving the assessment process at any time, and they were asked to sign an informed consent form. The assessment was completed in approximately 30–40 min.

### 2.4. Data Analysis

Participants were categorized into three groups based on CBI scores in order to analyse the relationship between caregiver burden and sleep quality in dependent people’s family caregivers. These groups were: (a) family caregivers with low and medium levels of perceived burden (CBI score ≤ 36) (Group 1); (b) family caregivers with high levels of perceived burden (CBI score > 36) (Group 2); and (c) non-caregivers controls, codified as no burden (control) (Group 3).

In the initial analysis, to compare the possible differences between the groups in several characteristics of the family caregivers and non-caregivers, of the care recipients and of the care situation, the data were expressed as frequencies and percentages for the categorical variables and as means and standard deviations for the continuous variables. The statistical analyses were carried out using the chi-square test (*χ*^2^) for categorical variables, and one-way analysis of variance (ANOVA) (*F*) and unpaired Student’s *t*-test (*t*) for continuous variables. The homogeneity of variances was tested through the Levene’s test (*L*).

Subsequently, a one-way multivariate analysis of variance (MANOVA) was performed, with the PSQI scores (7 component scores plus global score) serving as the dependent measures. Since this test assumes multivariate normality, this assumption was tested with Box’s M test. Later, a series of ANOVA were implemented for each of the dependent variables, and Scheffé’s post-hoc tests for multiple comparisons were conducted. A multiple regression analysis was finally accomplished to identify the significantly associated variables to sleep quality in family caregivers.

The statistical analyses were performed using IBM SPSS Statistics 20 (IBM Corp., Armonk, NY, USA). Results were considered statistically significant at the *p* < 0.05 significance level, and all tests were two-tailed.

## 3. Results

### 3.1. Characteristics of the Family Caregivers, the Care Recipients and the Care Situation

The sociodemographic characteristics of the family caregivers and the non-caregiver controls, including the results of statistical analyses, are summarized in Table 1. There were no significant differences between the groups in age, gender, marital status, educational level, and monthly incomes. Likewise, the two groups of family caregivers (groups 1 and 2) were similar with respect to “family relationship with the dependent”. Before these analyses, the homogeneity of variances was proven by Levene’s test (L_2,290_ = 0.26; *p* = 0.77).

Regarding the characteristics of the care recipients, there were no significant differences between the family caregiver groups neither in age nor in gender of the dependent person cared. However, significant differences were found in the cause of the dependence (*χ*^2^ = 13.99; *p* < 0.001). Physical disability was the cause of the dependence in 73.3% of the persons cared by the family caregivers of Group 1, compared to 47.0% of the care recipients attended by the caregivers of Group 2. By contrast, mental disorders were the cause of dependence in 53% of the persons cared for by the family caregivers of Group 2, compared to 26.7% of the care recipients attended by the caregivers of Group 1.

With respect to the care situation, there were no significant differences among family caregiver groups in the years providing care (14.27 years versus 14.64); nevertheless, there were significant differences in daily hours of care (*t* = −2.47; *p* < 0.05). The caregivers of Group 2 dedicated almost two more hours a day to the care of the dependent person (17.04 h versus 15.19 h).

### 3.2. Subjective Sleep Quality

Mean scores and standard deviations of the PSQI (total and subscales score) obtained in all the study groups are presented in Table 2. As seen, although poor sleep quality and pathological difficulties in this area were appreciated in all the groups, as reflected by mean PSQI, with total scores greater than five, poor sleep quality was much more pronounced in Group 2 (M = 10.43). In all the PSQI subscales, Group 2 scored higher than the other groups. The highest scores were obtained in sleep latency (M = 1.93), subjective sleep quality (M = 1.63) and sleep disturbances (M = 1.61); the lowest score was in use of sleep medications (M = 0.83).

**Figure 1:**
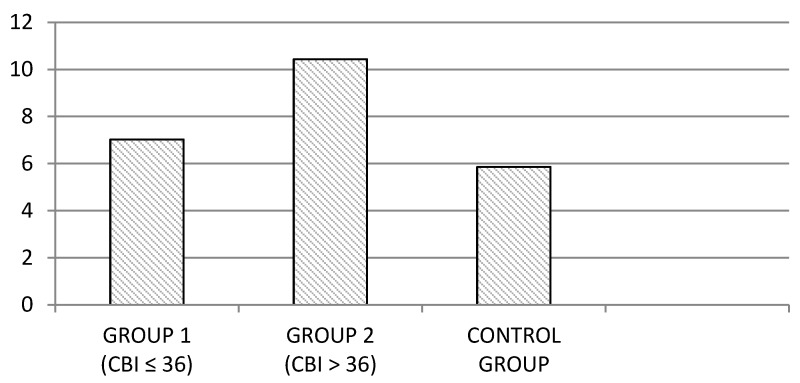
Mean scores of PSQI total in the family caregiver groups and non-caregiver controls.

Once compliance was secured with the relevant requirements (Box M = 55.69; *p* = 0.19), the MANOVA revealed statistically significant differences among the study groups (Wilks’s *λ* = 0.62; F_18,564_ = 8.46; *p* < 0.001). In view of this result, the differences among the groups for each dependent variable were analysed through one-way ANOVA. These analyses revealed that the differences among the groups were statistically significant in all the examined variables. Concretely, the group of family caregivers with high levels of perceived burden (Group 2) presented higher mean scores of PSQI total than those obtained by the other groups (see Figure 1). The differences between the groups in this dependent measure was significant (PSQI Total: F_2,290_ = 40.39; *p* < 0.001). In both cases, the Scheffé’s post-hoc test revealed differences among the following pairs: Group 1–Group 2; Group 2–Control Group; *p* < 0.05. There were no significant differences between Group 1 and Control Group.

**Figure 2:**
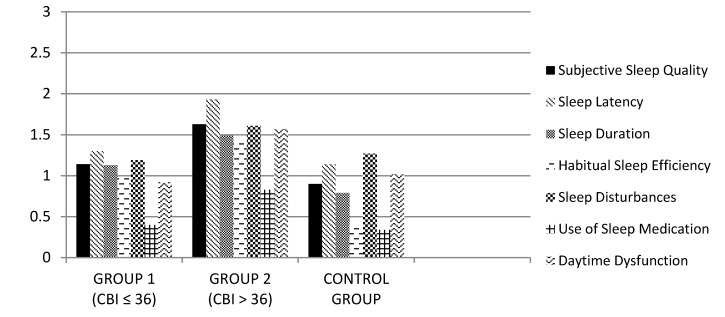
Mean scores of PSQI subscales in the family caregiver groups and non-caregiver controls.

Regarding the PSQI subscales (see Figure 2), there were significant differences among groups in subjective sleep quality (F_2,290_ = 25.55; *p* < 0.001), sleep latency (F_2,290_ = 16.99; *p* < 0.001), sleep disturbances (F_2,290_ = 14.90; *p* < 0.001), use of sleep medications (F_2,290_ = 6.94; *p* < 0.01) and daytime dysfunction (F_2,290_ = 20.12; *p* < 0.001). In all cases, the Scheffé’s post-hoc test revealed differences among Group 1–Group 2 and Group 2–Control Group (*p* < 0.05). There were no significant differences between Group 1 and Control Group.

On the other hand, there were also significant differences between groups in sleep duration (F_2,290_ = 18.34; *p* < 0.001) and habitual sleep efficiency (F_2,290_ = 24.24; *p* < 0.001). In both variables, the Scheffé’s post -hoc test revealed differences among all the pairs examined: Group 1–Group 2, Group 1–Control Group and Group 2–Control Group (*p* < 0.05).

### 3.3. Associated Variables to Sleep Quality in Family Caregivers

A multiple regression analysis was carried out with the PSQI total score as the dependent variable and all the characteristics of the family caregivers, of the care recipients, of the care situation and CBI score, as independent variables. Using the enter method, it was found that together the independent variables explained a significant amount of the variance in the extent of sleep quality F_12,188_ = 7.86; *p* < 0.001; *R*^2^ = 0.33; *R*^2^_Adjusted_ = 0.29). However, the daily hours of care (β = 0.19; *p* < 0.05) and the CBI score (β = 0.50; *p* < 0.001) were the only significantly associated variables with sleep quality in family caregivers.

## 4. Discussion

This study investigated the relationship between caregiver burden and sleep quality in dependent people’s family caregivers. According to our hypothesis, a direct relationship between caregiver burden and sleep quality was evidenced. 

Although a poor sleep quality and pathological difficulties in this area were appreciated in all the studied groups (both in the family caregiver groups and controls), as reflected by mean PSQI total scores greater than 5, the family caregivers with higher levels of perceived burden (Group 2) presented higher mean scores of PSQI total (a worse sleep quality). In fact, significant differences were found in this variable between this group and the other two groups. By contrast, no significant differences were found in PSQI total between the family caregivers with low and medium levels of perceived burden and the non-caregiver controls. This could be due either to the stress-related physiological processes that make sleep initiation more difficult or to intrusive thoughts at bedtime that could prevent a more burnt-out caregiver from being able to readily fall asleep.

Regarding the diverse PSQI components, Group 2 scored higher than the other groups in all subscales. There were significant differences between Group 2 and the other groups in all PSQI subscales: subjective sleep quality, sleep latency, sleep disturbances, use of sleep medications, daytime dysfunction, sleep duration, and habitual sleep efficiency. On the contrary, there were only differences between Group 1 and the Control Group in sleep duration and habitual sleep efficiency, but not in the other five subscales.

In sum, caregiver burden was significantly associated with poor sleep quality. This result is consistent with those obtained by Creese et al. [32], Peng, Lorenz and Chang [33] and von Känel et al. [34]. Poor sleep quality in family caregivers in addition to being an important risk factor, precipitates negative affective, behavioural and cognitive responses that include irritability, impaired and negative thoughts, decreased ability to concentrate or make decisions and poor motivation, which, in turn, can themselves increase caregiver burden. Thus, caregivers can develop a self-perpetuating spiral of sleep disturbances that is difficult to break and that can have adverse consequences for the health-related quality of life.

With respect to the characteristics of the family caregivers and controls subjects, of the care recipients and of the care situation, there were only differences in the cause of the dependence of the care receptor (physical disability “versus” mental disorder) and the daily hours dedicated to their care. The family caregivers with high levels of perceived burden cared for more people with mental disorders and less with physical disabilities than family caregivers with low and medium levels of perceived burden. Moreover, they dedicated more daily hours to the care of the dependent family member. Likely, caring for a dependent person due to the presence of a mental disorder is more challenging than caring for a dependent person due to the fact of a physical disability. In general, dependent people with mental disorders demand more supervision and attention, so that their care becomes a more stressful task and, therefore, comes with a greater risk of burnout and feelings of distress that can negatively affect the family caregiver’s health [35]. This finding is in line with previous studies that have noted that higher levels of care recipient behaviour problems were more consistently related to poor caregiver health [20]. However, these effects may be mediated, among other variables, by the link between the caregiver and the person cared, although this hypothesis must be contrasted in future studies.

A potential limitation of the present study is that, due to the large size of the sample studied, we did not perform an objective measurement of sleep quality using actigraphy or polysomnography. However, it is important to keep in mind that the components of sleep quality and their relevance vary according to the subject’s perception of the quality of their sleep and daytime functioning [36]; which is why self-reporting procedures are the assessment tools more usual in this research domain, particularly the PSQI, a well-validated instrument with appropriate psychometric properties. A second limitation was that our study did not collect more specific information about circadian rhythms and the sleep hygiene of family caregivers, which are important factors that can contribute to the development and maintenance of sleep problems in this collective [15]. Finally, another limitation was that the study included a cross-sectional methodology, which does not allow us to make causal inferences regarding the direction of associations. Moreover, there were differences in the assessment format between the groups of family caregivers and the control group; while the caregivers were assessed by trained psychologists, the control subjects were assessed through an online platform.

Despite these limitations, the findings of this study suggest the importance of developing effective and efficient preventive interventions for sleep disorders in the clinical practice in primary health care with family caregivers. For that reason, family caregivers need to be more specifically assessed to detect these health problems as early as possible through appropriate screening procedures.

## 5. Conclusions

The results of this study confirm the hypothesis initially proposed, allowing to conclude, at least in the conditions of this work, the existence of a direct relationship between caregiver burden and sleep quality. More specifically, this association is shown in a significant form in family caregivers with high levels of perceived burden compared with both non-caregiver controls and family caregivers with low and medium levels of perceived burden. Poor sleep quality is an important health risk factor so it is very important implement screening programs of sleep problems and concerns in the health primary care of family caregivers.

## Figures and Tables

**Table 1:** Characteristics of the family caregivers and non-caregiver controls, of the care recipients and of the care situation.

Characteristics	Group 1	Group 2	Control Group	Comparison
	(CBI ≤ 36)	(CBI > 36)		
***n* = 293**	***n* = 86**	***n* = 115**	***n* = 92**	
*Family caregivers/Non-caregiver controls*				
Age (years)	55.75 (9.64)	56.45 (10.39)	57.03 (11.03)	F_2,290_ = 0.34; *p* = 0.713
Gender				
Female	72 (83.7)	103 (89.6)	78 (84.8)	
Male	14 (16.3)	12 (10.4)	14 (15.2)	*χ*^2^ = 1.70; 2 *df*; *p* = 0.426
Marital status				
With partner	73 (84.9)	87 (75.6)	69 (75)	
Without partner	13 (15.1)	28 (24.4)	23 (25)	*χ*^2^ = 3.24; 2 *df*; *p* = 0.198
Educational level				
At least primary studies	73 (84.9)	101 (87.8)	79 (85.9)	
Without primary studies	13 (15.1)	14 (12.2)	13 (14.1)	*χ*^2^ = 0.39; 2 *df*; *p* = 0.824
Monthly incomes (Euros)				
<1000	14 (16.3)	28 (24.3)	18 (19.6)	
1000–2000	37 (43)	46 (40)	38 (41.3)	
>2000	7 (8.1)	11 (9.6)	10 (10.9)	
Do not know/No answer	28 (32.6)	30 (26.1)	26 (28.2)	*χ*^2^ = 4.43; 6 *df*; *p* = 0.817
Family relationship with the dependent (only family caregivers)				
Care recipient is the father/mother	35 (40.7)	53 (46.1)		
Care recipient is the son/daughter	19 (22.1)	20 (17.4)		
Care recipient is the partner or other	32 (37.2)	42 (36.5)		*χ*^2^ = 0.89; 2 *df*; *p* = 0.639
family member				
*Care recipient*				
Age (years)	68.31 (23.27)	74.01 (19.81)		*t* = −1.87; 199 *df*; *p* = 0.062
Gender				
Female	49 (57)	63 (54.8)		
Male	37 (43)	52 (45.2)		*χ*^2^ = 0.10; 1 *df*; *p* = 0.756
Cause of the dependence				
Physical disability	63 (73.3)	54 (47.0)		
Mental disorder	23 (26.7)	61 (53.0)		*χ^2^* = 13.99; 1 *df*; *p* < 0.001 *
*Care situation*				
Years providing care	14.27 (11.20)	14.64 (12.08)		*t* = −0.22; 199 *df*; *p* = 0.824
Daily hours of care	15.19 (5.85)	17.04 (4.72)		*t* = −2.47; 199 *df*; *p* < 0.05 *

*Note*: data are expressed as frequency (*n*) and percentage (*%*) for categorical variables and as mean ($\bar{X}$) and standard deviation (SD) for continuous variables. CBI: Caregiver Burden Inventory. * Significant difference.

**Table 2:** Mean scores (standard deviation) for PSQI (total and subscales score) in all the study groups.

Measures	Group 1	Group 2	Control Group	Tests of Between-Subjects Effects (F and *p*-Values)
	(CBI ≤ 36)	(CBI > 36)		
PSQI total	7.02 (3.43)	10.43 (4.27)	5.86 (3.57)	F_2,290_ = 40.39; *p* < 0.001 *
Subjective sleep quality	1.14 (0.69)	1.63 (0.80)	0.90 (0.73)	F_2,290_ = 25.55; *p* < 0.001 *
Sleep latency	1.30 (1.15)	1.93 (1.07)	1.14 (0.86)	F_2,290_ = 16.99; *p* < 0.001 *
Sleep duration	1.13 (0.97)	1.49 (0.83)	0.79 (0.65)	F_2,290_ = 18.34; *p* < 0.001 **
Habitual sleep efficiency	0.97 (1.06)	1.40 (1.19)	0.39 (0.77)	F_2,290_ = 24.24; *p* < 0.001 **
Sleep disturbances	1.19 (0.49)	1.61 (0.64)	1.27 (0.61)	F_2,290_ = 14.90; *p* < 0.001 *
Use of sleep medications	0.40 (0.97)	0.83 (1.22)	0.34 (0.90)	F_2,290_ = 6.94; *p* < 0.01 *
Daytime dysfunction	0.92 (0.78)	1.57 (0.81)	1.02 (0.77)	F_2,290_ = 20.12; *p* < 0.001 *

*Note*: all values are significant. * Scheffé’s post-hoc test: Group 1–Group 2; Group 2–Control Group; *p* < 0.05; ** Scheffé’s post-hoc test: Group 1–Group 2; Group 1–Control Group; Group 2–Control Group; *p* < 0.05. PSQI: Pittsburgh Sleep Quality Index.
